# Pattern of Splenectomy Indications in Kashan Shahid-Beheshti Hospital: A 5-Year Study

**DOI:** 10.5812/atr.8258

**Published:** 2013-02-01

**Authors:** Tahere Khamechian, Javad Alizargar, Maryam Farzanegan

**Affiliations:** 1 Department of Anatomical Sciences Research Center, Kashan University of Medical Sciences, Kashan, IR Iran; 2 Trauma Research Center, Kashan University of Medical Sciences, Kashan, IR Iran

**Keywords:** Elective Surgical Procedure, Splenectomy, Trauma

## Abstract

**Background:**

The spleen is amongst the most vulnerable organs which are easily injured in abdominal trauma. Nowadays, blunt trauma is the most prevalent indication of splenectomy.

**Objectives:**

We conducted this study to determine the pattern of indications for open splenectomies carried out during the past five years in Shahid-Beheshti hospital, a central hospital in Kashan, Iran.

**Patients and Methods:**

Demographic data of all patients who had undergone an open splenectomy in Kashan Shahid-Beheshti hospital during the past five years (2007-2012), indications for this type of surgery and outcome of patients were collected and entered into the study.

**Results:**

During the study period, the data from a total of 99 open splenectomies were entered in our study. Sixty-eight (68.7%) patients were male and 31 (31.1%) female. The mean age was 31.64 years, 75.8% of the cases had indications of trauma and 24.2% were elective. The most prevalent indication for trauma was motor-bike rider accidents and for elective ones portal hypertension.

**Conclusions:**

Most cases of splenectomy in our center caused by trauma, and among the different types of trauma, blunt trauma is the dominant cause. Road traffic accidents, in particular motor vehicle accidents, especially motorbike injures, are the most frequent cause of splenectomy. Due to the instability of trauma patients, a mortality rate of 8% seems to be reasonable for splenectomy.

We recommend that more research be conducted in this area and more cases enrolled with a reasonable follow-up period for splenectomy complications in this study.

## 1. Background

The spleen is amongst the most vulnerable organs which are easily injured in abdominal trauma. Nowadays, blunt trauma is the most prevalent indication for a splenectomy. Certain hematological diseases, tumors, and abscesses are other indications for this type of surgery. Open splenectomy (versus laparoscopic) is still the main surgical option for splenectomy in emergency situations as well as for many other indications. Although protected under the bony ribcage, the spleen remains amongst the most vulnerable organ easily injured in abdominal trauma. It is a friable and highly vascular organ holding 25% of the body’s lymphoid tissue and it has both hematological and immunological functions (1). Splenectomy roots can be tracked in history. The ancients used to remove the spleen from athletes in order to improve their wind (2). The first total splenectomy for disease is attributed to Adriano Zacarillo in 1549 (3). Nicolas Mathias is mentioned as performing the first total splenectomy for trauma in 1678, and the patient was reported to have survived for at least six years (3, 4). In the 1950's, splenectomy for splenomegaly with hypersplenism was the most common indication. But in the 1970's, splenectomy as part of a staging laparotomy for Hodgkin's lymphoma became the most common reason (5). With the introduction of sophisticated modern diagnostic and staging modalities, this indication became less common and splenectomy for trauma and hematological diseases emerged as the most common indication during the 1980's (6). In general, community practice the frequency of splenic rupture due to blunt trauma far exceeds that of penetrating trauma. Road traffic accidents, steering wheel injuries and seat belts are the most common causes implicated (4, 7). Penetrating injuries of the left thorax should arouse suspicion of splenic injury, as in such cases the frequency of associated intra-abdominal injuries is high (8). However, a splenectomy is generally required for trauma, some hematological diseases, tumors, abscesses, and hypersplenism, or as part of another surgical procedure (9). Overall, laparoscopic splenectomy has been gaining popularity as the treatment of choice for different benign and malignant diseases (10). Open splenectomy is still the main surgical option for splenectomy in emergency situations, as well as in many other instances, compared with laparoscopic one. Non-trauma indications for splenectomy and splenectomy procedures due to trauma are not well known.

## 2. Objectives

We conducted this study to determine the pattern of indications for open splenectomies carried out during the past five years in Shahid-Beheshti hospital, a central hospital in Kashan, Iran.

## 3. Patients and Methods

Demographic data of all patients who had undergone an open splenectomy in Kashan Shahid-Beheshti hospital during the past five years (2007-2012) and indications for this type of surgery were collected and entered into the study. Collected data consisted of age, sex, place of residence, reason for surgery, condition on admission, post-discharge mortality (if present) and its cause. During the study period, data from 99 patients who underwent a splenectomy was collected. Sixty-eight (68.7%) patients were male and 31 (31.1%) female and the mean age of the patients was 31.64 years. Approximately 75.8% of the surgeries had indications of trauma, 24.2% were elective, and 48% of the traumas injuries were due to motor vehicle accidents. Portal hypertension, metastasis, idiopathic thrombocytopenic purpura (ITP), and beta thalassemia were the most common indications for non-traumatic causes of splenectomy. Eight patients died after splenectomy. One patient had a pulmonary embolism, four with multiple trauma, two cases with intracranial hemorrhage, and one with a subarachnoid hemorrhage (SAH). Most causes of splenectomy in our center are the result of different types of trauma and among them blunt trauma is a dominant cause. Road traffic accidents, especially those involving motorbike injuries are the most frequent cause of splenectomy. Due to the instability of the trauma patients’ injuries, a mortality rate of 8% seems to be acceptable for splenectomy. The spleen was approached via an upper midline or left subcostal incision. None of the patients underwent a laparoscopic splenectomy. Patients requiring an elective splenectomy were given a vaccine prophylaxis against pneumococcal infection at least two weeks prior to their operation. Those who needed an emergency splenectomy for trauma were given a prophylaxis as soon as possible during the postoperative period. Postoperatively, patients were usually given amoxicillin 250 mg twice daily, as an antibiotic prophylaxis against postsplenectomy sepsis for at least the following two years after the procedure surgery. Data were entered into SPSS 14 software (SPSS Inc, Chicago, IL) and analyzed using descriptive statistics.

## 4. Results

During the study period, the data from a total of 99 open splenectomies were entered into our study. Sixty-eight (68.7%) patients were male and 31 (31.1%) female. The mean age of the patients was 31.64 years (SD = 19.45), ranging from 2 to 95. There were 47 (47.5%) cases from Kashan, 34 cases (34.3%) from rural villages, and 18 (18.2%) from other cities. In total, 75 surgeries (75.8%) had indications of trauma and 24 (24.2%) were elective. All surgeries were open ones. Among 75 trauma surgeries due to trauma, 8 (8.1%) were motor accidents (pedestrain), 28 (28.3%) motorbike accidents (injured rider). There were 19 surgeries (19.2%) due to car accidents, 8 (8.1%) heavy vehicle accidents, 11 (11.1%) falls from a height, 1 (1%) stab wound (stabbed with a knife during a quarrel) (Table 1). Five (5.1%) out of the 24 elective surgeries were due to idiopathic thrombocytopenic purpura (ITP), 3 (3%) beta thalassemia, 7 (7.1%) portal hypertension, 1 (1%) spherocytosis, 6 (6.1%) metastasis in the spleen, 1 (1%) hydatid cyst in the spleen and 1 (1%) was for a spleen abscess (Table 1). Thirty percent of the trauma patients were under 20 years-of-age and 26% of these young patients were 10 years or younger (Table 1). In total, 89 patients (89.9%) had stable vital signs on admission and 10 (10.1%) were unstable (Table 2), 91 patients were stable on discharge, but eight were not (Table 2). Death occurred in these eight patients after splenectomy. One had a pulmonary embolism, four with multiple traumas, two cases with intra-cranial hemorrhages, and one with a subarachnoid hemorrhage (SAH) (Table 3).

**Table 1.** Indications for Open Splenectomy and Age Group of Each Category

Indication	No. (%)	Under 20, y, No.	Under 10, y, No.	Total, No. (%)
**Trauma**				75 (75.8)
Motor-bike	8 (8.1)	2	0	
Motor-bike rider	28 (28.3)	8	0	
Car	19 (19.2)	8	4	
Heavy vehicle	8 (8.1)	3	1	
Fall	11 (11.1)	2	1	
Stabbing	1 (1)	0	0	
**Elective**				24 (24.2)
ITP a	5 (5.1)	0	0	
Beta thalassemia	3 (3)	3	0	
Portal Hypertension	7 (7.1)	3	1	
Spherocytosis	1 (1)	1	0	
Metastasis	6 (6.1)	1	0	
Hydatid cyst	1 (1)	0	0	
**Abscess**	1 (1)	0	0	

^a^Abbreviation: ITP, idiopathic thrombocytopenic purpura

**Table 2.** Number and Percentage of Unstable Patients Who Underwent Splenectomy Before and After Surgery

Indication	On Admission, No. (%)	On Discharge, No. (%)
**Trauma**	8 (10.7)	6 (8)
**Elective**	2 (8.3)	1 (4.2)
**Total **	10 (10)	7 (7)

**Table 3.** Number and Percentage of Mortalities After Splenectomy and Their Causes.

Indication	No. (%)	Total, No. (%)
**Trauma**		6 (75)
Pulmonary embolism	1 (12.5)	
Multiple trauma	4 (50)	
Intracranial hemorrhage	1 (12.5)	
**Elective**		2 (25)
Intracranial hemorrhage	1 (12.5)	
SAH a	1 (12.5)	
**Total**		8 (100)

^a^Abbreviation: SAH, subarachnoid hemorrhage

## 5. Discussion

In trauma patients, the spleen is the most commonly affected organ, followed by the liver and pancreas (11). In our center (during the study period), 75.8% of splenectomies were due to trauma. Out of these cases, only 1% was a penetrating trauma which took place during a quarrel, 74.8% blunt traumas, and 63.7% accident cases. In an investigation by Mufti et al. (1), only 36% of splenectomies were the result of accidents. This shows the higher accident rates (requiring the emergency interventions like splenectomy) in Kashan, Iran, than in Abbottabad, Pakistan. Meshikhes et al. (9) pointed to trauma with 20.8% of indications for splenectomy in Saudi Arabia. This indicates that they may have safer roads or more careful drivers and riders. In our center, motor accidents accounted for 36.4% of all splenectomies. Furthermore, if we compare the type of accident that leads to a splenectomy, motorbike accidents remain the dominant cause. These results are close to the 40% incidence of motor accidents that Akinkuolie et al. (12) described. Our results show that the most dangerous vehicle used in Kashan is the motorbike. Young adults (under 20) account for 28% of all motorbike rider accidents. This highlights the need for planning and education in this age group, to reduce the motorbike accident rates. There were 19.2% car accident trauma injuries in our study. It is worth mentioning that 42% of these patients were under 20 years, and half of these young victims were below 10 years-of-age. All of these young patients were travelling in the front seat with no seat belt. These findings point out the importance of placing children in the back seat and securing them with a seat belt. There was one stab wound and the patient remained stable before and after splenectomy and survived after an operation. The treatment of thalassemia major has traditionally included: 1) transfusion of red blood cells, 2) chelation, and 3) splenectomy. All three patients with beta thalassemia major were less than 20 years, stable on both admission and discharge, and survived after an operation. This indicates the safety of open splenectomy for these patients. Moreover, the size of the spleen is often greater than the small incision made for a laparoscopic splenectomy and this makes open splenectomy the only possible surgery option. Other studies have also confirmed the safety and necessity of open splenectomy for thalassemic patients (13). Splenectomy leads to improved red blood cell survival and it is also indicated for severe and probably moderate spherocytosis as well. There was a case of spherocytosis that was stable before and after splenectomy and survived after an operation. There is an increasing experience in many centers with the use of laparoscopic surgery for splenectomy, which has the advantage of a more rapid recovery, shorter hospital stay, and better cosmetic result. Nevertheless, the decision must rest on the surgeons, and it is dependent on their experience and the availability of suitable equipment (14). In our study 20% of non-traumatic indications for splenectomy were for an ITP. In a study conducted by Winslow et al. (15) this rate was 32%. There were five cases that underwent a splenectomy due to ITP in our study, three of whom survived, while two did not. One patient died of an intracranial hemorrhage, and another one due to a subarachnoid hemorrhage. Portal hypertension cases constituted 7.1% of our cases, whereas this indication involved 23% of splenectomies in the Meshikhes et al. (9) study. Most of the non-emergency cases in our city have the tendency to undergo the elective operations such as splenectomy (in portal hypertension) in the better equipped centers, and most of them probably refer to such centers. There were seven patients with portal hypertension who underwent splenectomy. No mortalities were reported in patients with metastasis, hydatid cysts or spleen abscesses. All of these patients were stable before and after an operation and survived. Some mechanisms of trauma may involve abdomen. For example motor bike riders and drivers may injure mainly in abdomen and this increases the prevalence of spleen injuries and splenectomies in these patients. We recommend that more research be conducted in this area and more cases enrolled with a reasonable follow-up period for splenectomy complications in this study.
